# The Treatment of Chronic Rheumatism

**Published:** 1872-04

**Authors:** 


### THE TREATMENT OF CHRONIC RHEUMATISM.

The therapeutics of this disease are reviewed at length by Dr. C. F. Ulrich, of Louisville, in the *American Practitioner*, June, 1871. He remarks:—

Assuming that rheumatism originates in defective assimilation, the indications of a cure are a correction of this difficulty, as well as the speedy and thorough elimination of the materies morbi.

One of the best remedies yet discovered for the accomplishment of this object is the ammoniated tincture of guaiacum, either alone or combined with bark. It acts as a stimulating evacuant, increasing the action of the skin, kidneys and bowels. Should it act too violently and produce diarrhœa, it must be controlled by opium.

Among the warm and stimulating remedies, we may name camphor, the oils of turpentine, cajeput, juniper, sassafras, and amber; the balsams of copaiba and Peru; aromatic and pungent plants, such as mustard, horse-radish, and arnica, montana, mezerion, seneca, dulcamara, etc. These may be used alone or combined with opium. The oil of turpentine and other terebinthinate substances will be found of specific value. But these substances, are sometimes nauseating to the patient, and producing stranguary when pushed beyond certain limits, should be combined with bark to obviate these disagreeable effects.

Sassafras has been found an excellent remedy in cases of languid constitution, with inactivity of the skin, kidneys, and bowels. It acts as a stimulant and sudorific, and possesses this advantage over guaiacum that it does not produce diarrhœa. It should always be accompanied by the free use of diluents to promote diuresis and diaphoresis. The same rule applies to the use of salines given as alternatives and eliminatives. Free dilution exercises a very important influence upon such remedies.

Sulphur has been found highly beneficial where the skin was inactive, given in half-drachm doses, accompanied with the sulphur vapor-bath. This, on account of its cheapness, has been a favorite remedy with the poor. It should, however, only be used in the muscular and neuralgic form of the disease. Colchicum is only useful when the disease approaches somewhat to the acute form, with inactive liver, swollen joints, effusion into the capsules, and where pain is aggravated by heat. In such cases it may be given with the alkalies and opium.

Iodide of potassium has been found by far the best remedy in the periosteal variety, seeming to exercise a special influence over the diseases of the periosteum. Many cases, which for a long time resisted all other remedies, have yielded to this drug. It should be given in combination with bark and sarsaparilla, and with opium at night. The bowels should be regulated by gentle laxatives, to overcome the natural intestinal torpor accompanying the disease, and to obviate the constipating effect of the opium. A valuable adjunct to the iodide of potassium is the daphne mezereon, which has been used by Sir Alex. Russell in venereal nodes. Being similar in its action to the iodide, it should only be used where that drug is indicated.

Cod-liver oil has been highly recommended by some; but of course it should only be used as an adjunct to other medicine in cases where there is emaciation. It has been used with excellent results in conjunction with liq. potass., iodid. potass., or with syr. ferri iodid.

Muriate of ammonia is an excellent remedy in some forms of rheumatism, especially the muscular. It acts as a stimulant to the bowels and skin and kidneys. Like most other remedies, it should be used in combination with bark. It has very little effect on periosteal and articular rheumatism. In some obstinate cases, where the system has been pervaded by the syphilitic virus, it is necessary to have recourse to mercury. The form that has been found most efficacious is the biniodide, given, of course, in combination with bark.

Opium, in some form or other, constitues one of the essentials in the treatment of chronic rheumatism ; not from any special curative power possessed by this drug, but because it imparts that rest of which the patient is deprived by the continual pain, and its nocturnal exacerbations.

But there are some cases in which the use of opium is inadmissible, either from idiosyncrasy or the peculiar condition of the patient. Yet relief must be afforded, and the sufferer must obtain the necessary rest, if we expect him to recover. It becomes our duty therefore to resort to some other hypnotic, as belladonna, stramonium, conium, or hyoscymus. Cannabis indica has been highly extolled by some, especially in cases of nervous exhaustion, on account of its exciting power, while others have derived no benefit whatever from it. Aconite has been found of great service in cases without inflammatory symptoms and without constitutional derangement, when pain and stiffness are the only trouble. But, owing to its poisonous properties, it should be administered with caution. Chloral hydrate in some cases has been found a very valuable substitute for opium.

In lumbago, with scanty and high-colored urine, bowels constipated, or evacuations dark and offensive, the disease is due in many cases to irritation of the lumbar and sacral nerves. In that case an active purgative and an enema of turpentine and castor-oil will relieve the pain by removing the pressure. This may be followed up by Rochelle salt and wine of colchicum. But when the above-named conditions do not exist, we may infer that the difficulty lies in the lumbar muscles, with their fasciæ and tendons. If there be symptoms of local congestion, cupping in the loins, alkaline baths, and alkaline fomentations are very useful. Where the symptoms of local congestion do not exist, and the complaint appears to be of a neuralgic character, electricity and galvanism may be employed to advantage. Stimulating embrocations followed by sedative fomentations, or friction followed by a flannel bandage, are frequently very beneficial. One of the most useful means of alleviation in lumbago and torticollis is that long used by the common people under the name of the ironing process. It consists in heating an ordinary flat-iron until it feels somewhat uncomfortably hot to the skin, and pressing it against the affected part, moving it about from place to place. An instrument invented by Dr. Day consists of a metal button, with an iron shank and wooden handle. This is heated and applied lightly and rapidly over the affected surface not being hot enough to blister it. This is styled the thermic treatment, in which its inventor professes unbounded confidence. Dr. James Arnott has a mode of treatment which is precisely the reverse of this, and consists in applying freezing mixtures to the part affected until local anæsthesia is produced. He claims entire success for this mode of treatment.

Of internal remedies, none occupy a higher rank than oil of turpentine, when the bowels are regular and the urine is clear; but it is useless when the reverse is the case. It should, however, be used with caution, on account of its liability to produce strangury, and its effects should be clasely watched.

The bath is a very important adjunct to other modes of treatment. The warm bath, with or without the addition of alkalies, the hot-air bath, and the vapor-bath, have all produced most beneficial results. To those whose condition enables them to bear cold, the shower-bath, followed by friction, is very beneficial. For localized affections, sometimes the cold douche is the best thing that can be done.

Among the local remedies, blisters have occupied the attention of the medical world for many years. Without much efficacy in muscular rheumatism or when the joints are affected, they have been found of immense value in the periosteal affection. This, in connection with the iodide of potassium, is by far the best treatment in that form of the disease. The compound iodine ointment is an excellent preparation for that purpose. The liniments and fomentation spoken of in lumbago and kindred affections are also extremely useful in old cases of joint disease.

Acupuncture has been highly spoken of in some cases of muscular rheumaiism. Mr. Churchill speaks of it in very favorable terms. Dr. Elliotson confirms his account of it. MM. Berlioz and Cloquet, of France, record many cases in which it has been successful.—*Half-Yearly Compendium,* Jan. 1872.

